# Left Paraduodenal Hernia: Case Report of Rare Cause of Recurrent Abdominal Pain

**DOI:** 10.7759/cureus.7156

**Published:** 2020-03-02

**Authors:** Sara Kadhem, Mohamed H Ali, Faisal H Al-dera, Noor A Alzayer, Hassan M Alyagoub

**Affiliations:** 1 Radiology, King Fahd Hospital of the University, College of Medicine, Imam Abdulrahman Bin Faisal University, Al-Khobar, SAU; 2 Surgery, King Fahad University Hospital, Dammam, SAU

**Keywords:** internal hernia, paraduodenal hernia, abdominal pain

## Abstract

Internal hernia is a relatively uncommon condition and is a rare type of intestinal obstruction. Paraduodenal hernia is considered the most common type of internal hernias. The rare prevalence and the variable symptoms make the clinical diagnosis of paraduodenal hernia a diagnostic challenge. We present the case of a 26-year-old male patient presented with a seven-day history of generalized intermittent crampy abdominal pain accompanied by nausea and multiple episodes of vomiting. He was otherwise healthy and had no history of previous abdominal operations. Computed tomography scan of the abdomen demonstrated sac-like clustered small bowel loops noted in the left upper quadrant, in the anterior pararenal space, consistent with the diagnosis of left paraduodenal hernia. Laparoscopic surgery for the repair of the hernia was planned but it was converted to open surgery due to technical difficulties. The patient tolerated the procedure without complications, and he was asymptomatic in the follow-up visit. This case sheds light on the importance of considering the diagnosis of left paraduodenal hernia in patients with recurrent abdominal pain, particularly among those who have not undergone abdominal surgery previously.

## Introduction

Internal hernia is an abnormal protrusion of abdominal viscera (most commonly small intestine or omentum) through a defect, which may be congenital or acquired, within the peritoneum or mesentery. Internal hernia is a relatively uncommon condition accounting for less than 1% of the abdominal hernias, and it is a rare cause of intestinal obstructions [1]. The types of internal hernia (in decreasing order) are paraduodenal, pericecal, foramen of Winslow, transmesenteric and transmesocolic, pelvic, intersigmoid, retroanastomotic, and transomental hernia [1]. Paraduodenal hernia is difficult to diagnose because of variable clinical presentation which may include acute intestinal obstruction and recurrent abdominal pain [2]. Herein, we present a case of a young male patient who presented with symptoms consistent with incomplete intestinal obstruction; a subsequent computed tomography (CT) scan demonstrated sac-like clustered small bowel loops noted in the anterior pararenal space consistent with left paraduodenal hernia. Subsequently, the patient underwent a laparoscopic attempt for a reduction of the hernia that was converted to open surgery due to technical difficulties. The entrapped intestinal loops were reduced. The patient tolerated the procedure well without complications.

## Case presentation

A 26-year-old male patient presented to the emergency department with a seven-day history of generalized intermittent crampy abdominal pain. The pain was accompanied by nausea and multiple episodes of vomiting. The pain worsened after meals. His last bowel motion was three days before the presentation; however, he continued to pass flatus. He had multiple similar episodes of this pain over the last two months that resolved spontaneously. He was otherwise healthy and had no history of previous abdominal operations.

On examination, she was in pain and her vital signs were as follows: pulse rate as 90 bpm, blood pressure of 121/76 mmHg, and respiratory rate of 20 bpm, and she was afebrile. Abdominal examination revealed a distended abdomen, with no tenderness or guarding. There was hyperactive bowel sounds all over the abdomen. No palpable masses or organomegaly and no external hernias were noted. The digital rectal examination was unremarkable.

**Figure 1:**
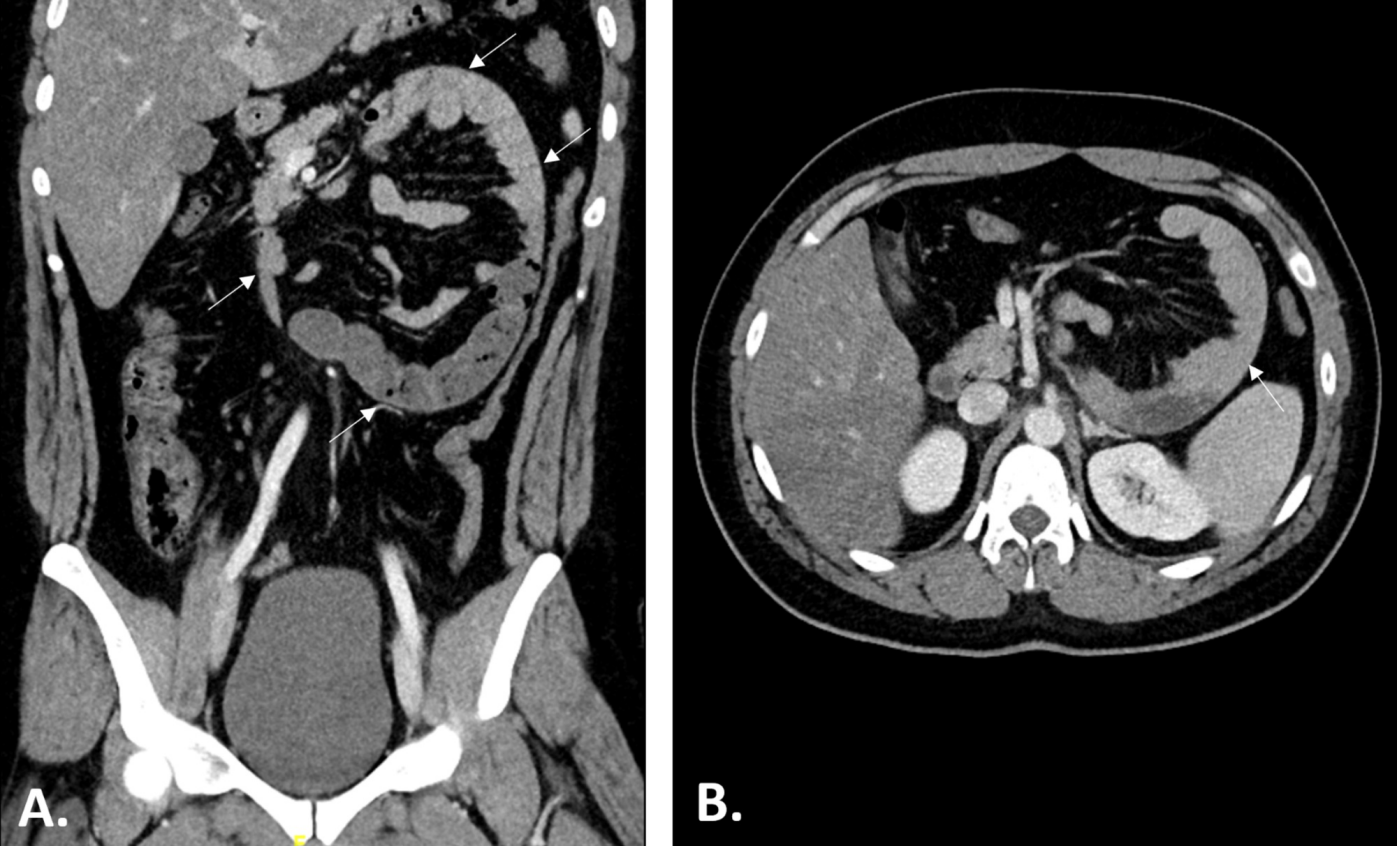
CT of the Abdomen (A: Coronal and B: Axial Images) CT images of the abdomen demonstrating sac-like clustered small bowel loops (arrows) noted in the left upper quadrant, in the anterior pararenal space. Also note that the remainder of large bowel is completely collapsed. These findings are suggestive of the diagnosis of left paraduodenal hernia.

Laboratory studies showed hemoglobin of 13 g/dL, white blood cell count of 8,000/mL shift, and platelet count of 144,000/mL. Serum electrolytes, urea, creatinine, and liver function tests were all within normal limits. The chest X-ray did not reveal any abnormalities with no free air under the diaphragm. CT scan of the abdomen was performed (Figure 1), which demonstrated sac-like clustered small bowel loops noted in the left upper quadrant, in the anterior pararenal space. The remainder of the large bowel is completely collapsed. These findings were suggestive of the diagnosis of left paraduodenal hernia, and the surgical team was then informed.

Given the aforementioned clinical and radiological findings, the patient was then prepared for diagnostic laparoscopy, which had confirmed the diagnosis of a left paraduodenal hernia. The surgery was performed under general anesthesia with the patient placed in a supine position, and three ports were inserted to carry out the procedure. After establishing the pneumoperitoneum and introducing trocars, careful adhesiolysis was performed. The entrapped bowel loops were noted, but it was difficult to reduce laparoscopically because of the dense adhesions. The surgery was then converted to open, and enterolysis was performed. The patient tolerated the procedure well with no complications and was discharged on the fourth postoperative day with no active complaints during the follow-up visit.

## Discussion

Paraduodenal hernia, also known as mesocolic hernia, was first described at autopsy by Neubauer in 1786 [3]. Later, an accurate scientific description of the condition was provided by Treitz in 1857, who considered it a retroperitoneal protrusion of abdominal viscera [3]. In 1889, the classification of hernia into the distinct left and right types was made by Jonnesco [4].

There are multiple theories about the mechanism of paraduodenal hernia formation. The most widely accepted theory was first described by Andrews in 1923, who postulated that it results from an embryological error during the midgut rotation. The failure of the mesentery to fuse with the parietal peritoneum of the posterior abdominal wall after the return of the herniated intestinal loops to the abdominal cavity in the early weeks of development creates a potential space of herniation behind the mesocolon [5].

Left paraduodenal hernia is about three times more common than its right counterpart (Waldeyer’s hernia). It arises from the fossa of Landzert, which is present in 2% of the population, through a defect in the left portion of the transverse mesocolon causing retroperitoneal retrocolic herniation of small intestine (usually proximal jejunum). The fossa of Landzert is located to the left of the fourth part of the duodenum, posterior to the inferior mesenteric vein and ascending branch of a left colic artery, where they form the free edge of hernia, and directly below the posterior parietal peritoneum [1].

The rare prevalence and the variable symptoms make the clinical diagnosis of paraduodenal hernia a diagnostic challenge. The clinical presentation is entirely non-specific. It ranges from being completely asymptomatic and found incidentally during surgery or autopsy to acute intestinal obstruction seen in 50% cases with the risk of gangrene and perforation [6,7]. Such a myriad of clinical features often lead physicians to misdiagnose paraduodenal hernia as biliary disease or peptic ulcer resulting in patients receiving unnecessary therapeutic interventions.

The first case of paraduodenal hernia that had a correct preoperative diagnosis was made by Kummer in 1921 by the barium study [8]. Currently, the CT scans superseded the upper gastrointestinal contrast studies as the abdominal CT scan became the diagnostic modality of choice. The CT findings show a smooth encapsulated sac-like mass of the small intestinal loops between the stomach and the pancreas at the level of the ligament of Treitz. Additionally, the displacement of mesenteric vessels may be apparent [1].

Once diagnosed, the surgical treatment of paraduodenal hernia is indicated because the lifetime risk of incarceration or strangulation is over 50% with a mortality risk of 20%-50% [9]. The same principles of hernia surgery apply in treatment of paraduodenal hernia including reduction of the hernia content, resection of the necrotic intestinal segment, if any, and repair of hernial orifice by closure or wide opening, by performing an incision along the avascular plane of mesocolon or doing division of the inferior mesenteric vein, so that the hernia sac becomes part of the peritoneal cavity.

The surgical treatment of the paraduodenal hernia may be carried out by the conventional open approach or minimally invasive laparoscopic approach. In our case, because of the dense adhesion, it was converted to open surgery.

## Conclusions

The diagnosis of internal hernia should be taken into consideration in patients with recurrent abdominal pain, particularly among those who have not undergone abdominal surgery previously.
